# A retrospective study on the impact of bar flipping on the recurrence of pectus excavatum after the Nuss procedure

**DOI:** 10.1186/s13019-021-01621-9

**Published:** 2021-08-28

**Authors:** Yu-Jiun Fan, Po-Cheng Lo, Yuan-Yu Hsu, I-Shiang Tzeng, Bo-Chun Wei, Yeung-Leung Cheng

**Affiliations:** 1 Division of Thoracic Surgery, Department of Surgery, Taipei Tzu Chi Hospital, Buddhist Tzu Chi Medical Foundation, New Taipei City, No. 289, Jianguo Road, Xindian District, 231 Taiwan; 2 Department of Radiology, Taipei Tzu Chi Hospital, Buddhist Tzu Chi Medical Foundation, New Taipei City, Taiwan; 3 Department of Research, Taipei Tzu Chi Hospital, Buddhist Tzu Chi Medical Foundation, New Taipei City, Taiwan; 4 School of Medicine, Tzu Chi University, Hualien, Taiwan

**Keywords:** Pectus excavatum, Nuss procedure, Bar flipping, Recurrence

## Abstract

**Background:**

The Nuss procedure is widely used to correct pectus excavatum. Bar displacement is a common complication associated with this procedure. How the flipping of the bar affects pectus excavatum recurrence has not been reported. In our study, we discuss this and also offer an easier method to determine bar flipping.

**Methods:**

This retrospective study analyzed pectus excavatum patients who underwent primary Nuss repair from August 2014 to December 2018. The preoperative and postoperative Haller indices were measured on chest radiographs (*cxr*HI). The slope angle of bar flipping (α) was measured on lateral chest radiographs. The improvement index after surgical repair was calculated by: ([preoperative *cxr*HI-postoperative *cxr*HI]/preoperative *cxr*HI × 100). The impact of α on the improvement index was analyzed using one-way analysis of variance and receiver operating characteristic tests.

**Results:**

In this study, 359 adult and adolescent patients with an average age of 23.9 ± 7.7 years were included. We formed four subgroups based on the α value: α ≤ 10° (n = 131), α = 11–20° (n = 154), α = 21–30° (n = 51), and α > 30° (n = 23). The mean improvement indices in these groups were 27%, 28%, 26%, and 13%, respectively. Patients with α > 30° were associated with a significantly poorer improvement index than those from the other subgroups (*p* < 0.001).

**Conclusions:**

The α value is an alternative measurement method for presenting the radiological outcomes after the Nuss procedure. An α > 30° indicates a possible recurrence of pectus excavatum after the Nuss repair. Surgical revision may be considered in patients with an α > 30°, while monitoring should be considered in the other patient groups.

## Introduction

Pectus excavatum (PE), also known as funnel chest, is a structural deformity in which the sternum sinks in the center of the anterior chest wall. The exact cause of PE remains unknown. This congenital problem may become more severe in adolescence, as the chest wall deformation may be aggravated by an overgrowth of the costal cartilage. The estimated prevalence of PE in the adult population is 1 in 250 individuals [1]. Patients with PE do have not only a negative body image but also experience physical problems such as chest pain, exercise intolerance, dyspnea, sleep apnea, and rapid heart rate due to the compressive effects on the heart and lungs [2]. The Nuss procedure, first reported in 1988 by Nuss et al., is a minimally invasive method widely used to correct PE. In this method, metal bars are placed retrosternally, with the lateral ribs acting as hinge points, to lift the depressed chest wall.

Despite the excellent outcomes, a series of outcome studies have revealed that bar migration and PE recurrence are the major problems associated with the Nuss procedure [3–6]. The reported bar migration rates vary [5–7]. Previous investigators have classified the mechanisms of bar migration into bar flipping (bar rotates), lateral sliding (bar slides horizontally to one side), and hinge-point disruption (bar shifts dorsally) [7]. According to our clinical observation, PE recurrence is most likely associated with bar flipping (to some degree) rather than lateral sliding. However, no study has investigated the association between bar flipping and outcomes of the Nuss procedure. Therefore, this study aimed to identify the association between the degree of bar flipping and PE recurrence. Besides, there is still no standard consensus on the measurement of the bar flipping degree. We designed a method to measure the slope of the bar and to estimate PE recurrence. Our study provides a guideline to survey and follow patients who may warrant revision surgery after the Nuss procedure.

## Methods

**Fig. 1:**
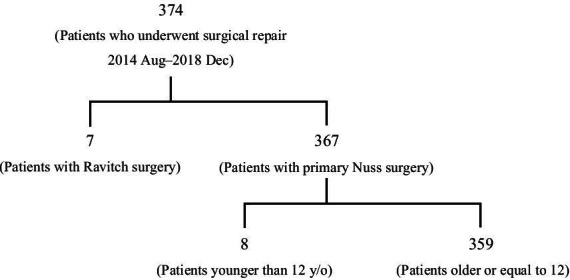
Flow chart for the inclusion of patients in the study

Between August 2014 and December 2018, 374 patients underwent a primary repair for PE at the Taipei Chi-Tzu Hospital in New Taipei City, Taiwan. Of these, 367 patients received the Nuss repair, while seven received the Ravitch repair. Of the 367 patients who underwent the Nuss procedure, 359 of which were adolescents and adults above 12 years of age and they were included in this retrospective analysis (Fig. 1). This retrospective study was approved by the Ethics Committee and the Institutional Review Board of the Taipei Tzu Chi Hospital, Taipei, Taiwan, ROC (IRB No: 08-X-101). Patient consent requirement was waived by the institutional review board due to the study’s retrospective nature.

All surgeries were performed by a single surgeon (Dr. Cheng); the surgical technique followed has been described previously [8]. The indications for surgery were based on the criteria established by Nuss and Kelly [9]. Patients’ baseline characteristics, including sex, age, body weight, height, preoperative Haller index, and/or the presence of scoliosis, were recorded as preoperative assessments. Further preoperative examinations included chest radiography, electrocardiography, pulmonary function test, echocardiography, and chest computed tomography (CT). The Haller index (HI) [10], which is the ratio of the maximum transverse thoracic diameter to the minimum sternal-to-anterior vertebral body distance on chest CT, was calculated on anteroposterior and lateral views of chest radiographs instead (cxrHI) (Fig. 1). The preoperative and postoperative cxrHI could be used to estimate the preoperative severity of PE and postoperative outcomes [11, 12]. Because poor improvement in PE after surgery can indicate PE recurrence, we used the change of cxrHI ($\Delta$ *cxr*HI) (i.e., the improvement index) to estimate the degree of PE recurrence in an objective way using the following formula: [(preoperative *cxr*HI-postoperative *cxr*HI)/preoperative *cxr*HI] × 100.

**Fig. 2:**
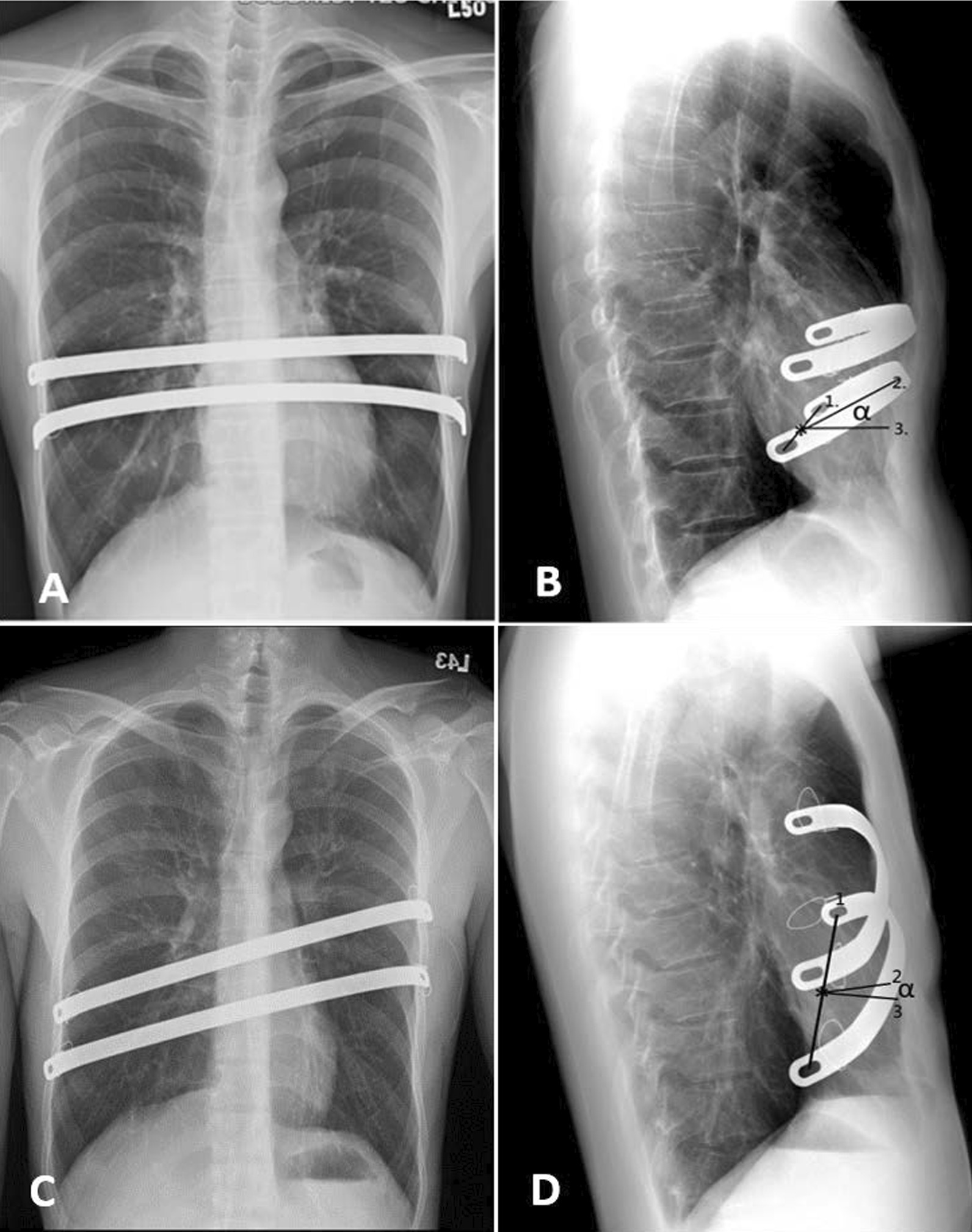
Measurement of the slope angle (α) of bar flipping: α is the angle between Line 2 and Line 3; Line 1 connects the two end holes, and the star sign marks the midpoint of Line 1, while Line 2 represents the star sign to the dorm of the bar. Line 3 represents the star sign where the optimal dorm location should be. This measurement method can be applied to bars inserted either horizontally (**A**, **B**) or obliquely (**C**, **D**)

We also designed an easier method for measuring the degree of bar flipping by using the slope angle (α). On lateral chest radiography, α was measured as the angle between the two lines connecting the midpoint of the two end holes of the bar to 1) the uppermost part of the arch of the bent bar and 2) the expected point of the dorm’s optimal position (Fig. 2). The HI and the α on the chest radiograph were measured by an experienced surgeon and a radiologist, who were both blinded to the clinical data.

### Statistical analyses

**Fig. 3:**
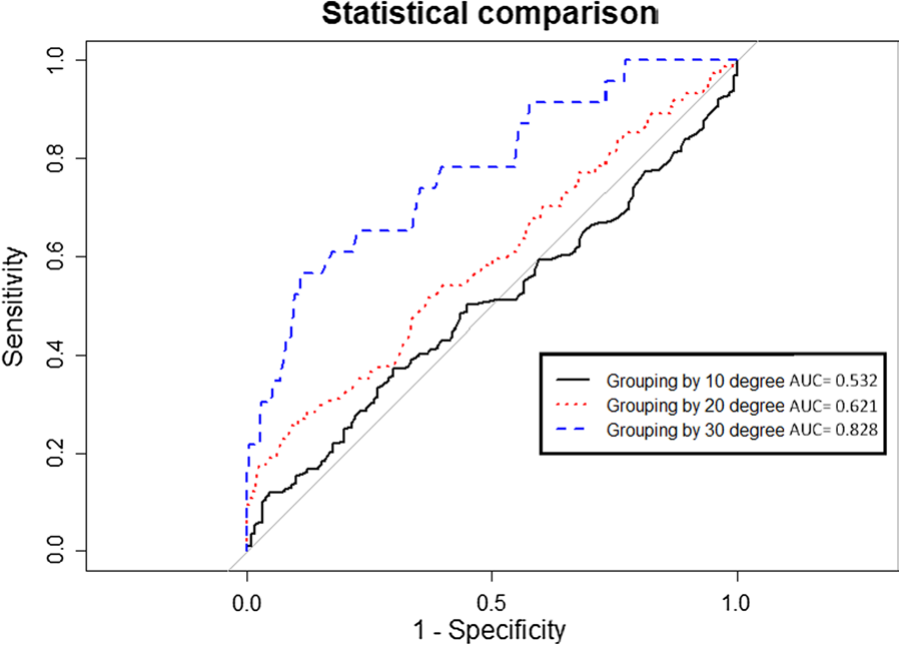
The DeLong test shows that grouping by 10° and grouping by 20° are not significant (*p* = 0.061). Grouping by 20° and 30° (*p* = 0.006) and grouping by 10° and 30° (*p* < 0.001) are significant

Statistical analyses were performed using IBM SPSS Statistics for Windows, version 24 (IBM Corp., Armonk, NY, USA). The Kolmogorov–Smirnov test revealed that all investigated parameters in our study were distributed normally. Continuous data were expressed as means ± standard deviations, while categorical data were expressed as counts (%). The optimal cuff-off value for the angle of bar flipping was determined by receiver operating characteristic (ROC) curve analysis. Based on the α of the most severe bar flipping, the patients were categorized into four groups, namely, α ≤ 10°, α = 11–20°, α = 21–30°, and α > 30°. The improvement indices were compared between these groups using one-way analysis of variance (ANOVA), and the Scheffe test was used as a post hoc test. Using the cut-off value at α = 30°, the differences between the groups were analyzed using a two-sample *t*-test for continuous data and the chi-square test for categorical data. ROC curves were created using varying values for α as the categorization criteria and the I index to compare the best area under the curve (AUC) value. The DeLong test was further used to compare the C-statistic between different α values (Fig. 3) [13]. ROC curve comparisons were conducted using R version 4.0.0 (pROC package). A *p* value of < 0.05 was considered statistically significant.

## Results

The study group comprised 304 men and 55 women with an average age of 23.9 ± 7.7 years. The mean postoperative follow-up period was 2.2 ± 1.3 years, while no individual follow-up was of < 6 months. The α ≤ 10°, α = 11–20°, α = 21–30°, and α > 30° groups comprised 131, 154, 51, and 23 patients, respectively (Table 1). The improvement indices in the α ≤ 10°, α = 11–20°, α = 21–30°, and α > 30° groups were 27% [95% confidence interval (CI): 17–37%], 28% (95% CI: 16–40%), 26% (95% CI: 14–38%), and 13% (95% CI: 4–22%), respectively (Table 1). The higher the improvement index, the more modest was the postoperative PE improvement. One-way ANOVA and post hoc analysis of the improvement indices within the groups revealed that an α > 30° was associated with a decreased improvement index, indicating a low clinical improvement (*p* < 0.001). There were no significant intergroup differences in the improvement indices when α ≤ 30° (Table 2). ROC curve analysis, based on a grouping by an α of 10°, 20°, and 30°, revealed that grouping by 30° achieved the best AUC (0.828). Hence, grouping by α = 30° achieved excellent discrimination, while grouping by α = 10° (AUC = 0.532) or α = 20° (AUC = 0.621) achieved poor discrimination. The DeLong test for the three ROC curves also revealed that the AUC, when grouped by α = 30°, was significantly different from the AUCs when grouped by α = 10° or α = 20°; however, there were no significant differences in the AUCs when grouped by α = 10° and α = 20° (Fig. 3). Univariate analysis revealed that α > 30° was associated with significantly higher body weight and higher HI values. However, there were no significant differences in age at repair, the number of bars, follow-up period, sex, scoliosis, or asymmetric body features between patients with α > 30° and with α ≤ 30° (Table 3). Furthermore, 7 of 23 patients in the α > 30° group voluntarily underwent a revision Nuss procedure after the comprehensive evaluation of significant recurrence and symptoms. These seven patients comprised five men and two women, and the mean age was 25 ± 8.2 years. The median [interquartile range (IQR)] period between the revision surgery and the first operation was 49 days (IQR: 27–71 days). Patients who underwent the revision Nuss procedure experienced a significantly greater improvement in the α and cxrHI values than after the first surgery (*p* < 0.05) (Table 4).

**Table 1:** Descriptive statistics of the different groups

Flipping angle	N	cxrHIpre^a^	cxrHIpost^b^	ΔcxrHI^c^	I index^d^
≤10°	131	3.69 ± 0.7	2.66 ± 0.4	1.03 ± 0.57	27 ± 10
11–20°	154	3.95 ± 0.8	2.77 ± 0.4	1.18 ± 0.77	28 ± 12
21–30°	51	3.94 ± 0.9	2.86 ± 0.4	1.09 ± 0.7	26 ± 12
> 30°	23	3.96 ± 1.5	3.46 ± 1.3	0.5 ± 0.5	13 ± 9

^a^Haller index on the preoperative chest radiograph

^b^Haller index on the postoperative chest radiograph

^c^cxrHIpre − cxrHIpost

^d^improvement index = (cxrHIpre − cxrHIpost)/cxrHIpre

**Table 2:** Multiple comparisons between the four groups (Scheffe post hoc test)

(I) Group	(J) Group	Mean Dif (I − J)	*p*-value
≤ 10°	11–20° 21–30°	− 0.012 0.009	0.850 0.974
	> 30°^†^	0.136	** < 0.001**
	≤ 10°	0.012	0.850
11–20°	21–30°	0.02	0.732
	> 30°^†^	0.148	** < 0.001**
	≤ 10°	− 0.009	0.974
21–30°	11–20°	− 0.02	0.732
	> 30°^†^	0.127	** < 0.001**

^†^There is a statistical difference between group > 30° and other groups (*p* < 0.001)

*p* < 0.05 are given in bold

**Table 3:** Comparison of the clinical characteristics based on a grouping by 30°

	α ≤ 30° (n = 336)	α > 30° (n = 23)	*p*-value
Age at Nuss repair, years (mean ± SD)	23.8 ± 7.8	25 ± 6.8	0.409
Sex, n (%)			0.754
Male	284 (85%)	20 (87%)	
Female	52 (15%)	3 (13%)	
Flipping angle, degrees (mean ± SD)	12.8 ± 6.3	40.4 ± 5.8	** < 0.001**
Body weight, kg (mean ± SD)	57.6 ± 10.7	63.6 ± 11.5	**0.01**
Haller index, (mean ± SD)	4 ± 0.9	4.4 ± 1.8	**0.018**
Observation period, years (mean ± SD)	2.2 ± 1.3	1.9 ± 1.6	0.405
BarN^†^, n (%)			0.641
1	33 (10%)	2 (9%)	
2	269 (80%)	20 (87%)	
3	34 (10%)	1 (4%)	
Scoliosis, n, (%)			0.596
Yes	316 (94%)	21 (91%)	
No	20 (6%)	2 (9%)	
Symmetry n, (%)			0.593
Yes	156 (46%)	12 (52%)	
No	180 (54%)	11 (48%)	

^†^BarN: the number of inserted bars in each patient

*p* < 0.05 are given in bold

**Table 4:** Comparison of the slope angle and the Haller index in seven patients before and after the revision Nuss surgery

	Before revision surgery	After revision surgery	*p* value
Slope angle α (IQR), degrees	39.6 (38.5–52)	10.4 (9.1–23.7)	**0.018**
cxrHI (IQR)	3.5 (3.1–3.8)	2.9 (2.5–3.1)	**0.043**

There was a significant difference (*p* < 0.05) in the α and HI before and after the revision Nuss procedure

*p* < 0.05 are given in bold

## Discussion

Minimally invasive repair of pectus excavatum (MIRPE), Nuss procedure, specially modified to be performed with a thoracoscopic aid [8] being used to correct pectus excavatum has shown satisfactory long-term results along with a better quality of life in over 90% cases who underwent primary repair, similar to the results of our clinical study[6, 14, 15]. The most common complication of the Nuss procedure is bar placement [16]. In our study, we used the lateral fixation method bilaterally with wire or stabilizers. The majority of the bar displacements in our cases were due to bar flipping, although we did encounter a few inconspicuous lateral migration and undetectable dorsal shift migration cases that could be disregarded. Hoksch et al. reported that in their analysis of 129 cases, 9 patients (7.0%) required surgical revision due to bar displacement [15]. Kelly et al. which reported by far the biggest case analysis with 1215 patients who underwent minimally invasive repair, reported that 4% of their cases had postoperative complications of the displacement of bars which required surgical revision [6]. Nevertheless, PE recurrence remains the most important unfavorable outcome of the Nuss repair. Previous studies have reported that the risk factors for PE recurrence after the Nuss procedure include surgical complications, younger age, earlier bar removal, higher HI, and higher body weight [3–6]. Contrary to other study that focused on PE recurrence after bars removal, our study discusses PE recurrence before bar removal [12]. We believe that bar flipping affects PE recurrence to some extent; however, in our clinical observation on postoperative follow-up chest radiography, in some cases, there was no obvious chest wall recession despite signs of bar flipping. Few studies have defined the bar displacement, or proposed how the displacement affects the operation revision. Moreover, the degree of bar displacement and the method to measure it are still inconsistent and seldomly mentioned. Kelly et al. reported that a bar movement more than fifteen degree is indicative for operative repositioning [6]. Cho et al. defined bar displacement more than twenty degree as severe bar migration [12]. However, in our study, no statistical differences in PE improvement were observed when the flipping of bars is less than 30°. We presumed that one of the reason causes the discrepancy of angle between the above studies and ours may be due to a difference in the method to determine bar flipping. Moreover, the authors of the above two studies have not set the reference point when mentioned the “bar movement”. Furthermore, Cho et al. used only one arm of the bar to measure the flipping angle which may change due to slight changes in the shooting angle of the follow-up plain film and the author didn’t mention how to measure when the bar inserted obliquely in certain cases. To diminish the deviation, we chose the middle point of the two arms as the reference point (Fig. 2). Similar to Cho’s study, we also considered the optimal point of the dorm of the bar to be where the force vector application was perpendicular to the sternum. Different measurement tools, CT or plain film, may also influence the assessment of migration. We used a lateral view of chest radiography to determine the bar migration as well as PE recurrence by calculating the change in postoperative Haller index, as described in previous study [11, 12]. They have indicated a significant correlation between Haller index using chest CT and simple radiographic data.

In most of the reports, the recurrence of PE has been simply identified by re-cave-in appearance and patient’s symptoms [3–6, 12]. The distinction between our work and the others is that we did not consider bar migration and PE recurrence as “yes- or -no” events. We considered the bar’s flipping and the degree of PE recurrence as “continuous variables”. In our study, we used an improvement index to quantify PE recurrence. Since poor improvement in PE after surgery can indicate PE recurrence, we used ΔcxrHI, the change between pre- and postoperative cxrHI (i.e., the improvement index) to objectively estimate the degree of PE recurrence. A significant factor that influences the improvement index was a flipping angle of more than 30°. Sa et al. designed an original method to measure bar migration in which they calculated the distance from the sternal angle to the upper border of the metal bar on the lateral chest radiograph to represent the bar’s migration. They had 61 patients who underwent the primary Nuss procedure, of whom 7 (11%) required reoperation for bar displacement. By standardizing the distance of migration (D_0_ − Dx/D_0_ × 100), they developed a bar displacement index (BDI) and found that the optimal cut-off value of BDI warranting reoperation was 8.7 [17].

In our previous study, postoperative changes in chest wall shapes mostly occurred within one month, and the chest wall diameter stabilized three months after the Nuss repair [18]. All cases in our present study were followed up for at least six months, which was an adequate time period for monitoring PE recurrence. A correlation between bar flipping and PE recurrence can be explained as follows: The convex bars strut the depressed sternum, and the tendency of the anterior chest wall to rebound inwards causes bar flipping even before the chest wall can be remodeled. The bar still has some strength when the flip angle is less than thirty degree. A tilted bar lacks support, which may cause PE recurrence; therefore, reciprocal causation is suggested between bar flipping and PE recurrence. Our study shows that the bar number does not influence postoperative bar flipping or the PE recurrence rate. This may be because the number of bars placed is decided by an immediate intraoperative outcome which means we would insert additional bars if one did not have enough supporting strength for lifting the concave chest wall. Although twenty-three of our patients with bar flipping angle more than thirty degree had PE recurrence, only seven of them underwent a revision Nuss procedure after evaluating aspects such as the severity of recurrence and the patient’s willingness and symptoms. For patients who underwent a revision repair for PE recurrence, the Nuss procedure remains an adequate choice with good outcomes, regardless of whether the initial repair was also a Nuss procedure; this is consistent with the results from Casamassima et al. [19] We hypothesize that the repositioning of the Nuss bars in a revision procedure increases the likelihood of successfully lifting the depressed anterior chest wall and provides additional time for chest wall remodeling.

We acknowledge that this study was retrospective and was performed at a single institute. Our case numbers with a bar flipping angle more than thirty degree and revision cases were limited. Researchers are encouraged to use our test model to conduct more clinical studies and provide solid evidence on PE recurrence. Moreover, a longer follow-up period is still required for patients who undergo a revision Nuss procedure.

## Conclusions

PE recurrence is the biggest concern in patients undergoing the Nuss procedure. In this study, the patients comprised adults who underwent an initial surgical PE repair. We introduced a simple and effective method for determining bar flipping. Our findings indicate that a bar flipping angle more than thirty degree is a predictor of PE recurrence and may require revision surgery. Our study’s limitations include its single-center, retrospective nature, and the fact that only one thoracic surgeon performed all surgeries. More cases are still required to support our findings. The Nuss procedure is an adequate follow-up intervention in patients with PE recurrence who underwent the Nuss procedure as the primary repair; however, a long-term follow-up is still required for such patients.

## Data Availability

The data sets used and analyzed during the current study are available from the corresponding author.

## Abbreviations

ANOVA: One-way analysis of variance
AUC: Area under the curve
CI: Confidence interval
CT: Computed tomography
cxrHI: Chest X-ray HI
HI: Haller index
IQR: Interquartile range
Postop cxrHI: Postoperative cxrHI
Preop cxrHI: Preoperative cxrHI
PE: Pectus excavatum
ROC: Receiver operating characteristics
